# Supplementary material for: Chemical genetic identification of CDKL5 substrates reveals its role in neuronal microtubule dynamics
Source: EMBO J. 2018 Sep 28;37(24):e99763. doi: 10.15252/embj.201899763 (PMC6293278; doi:10.15252/embj.201899763)

Table of Contents

Appendix figure legends	p. 2
Appendix Figure S1 - Analog-sensitive CDKL5 phosphorylates AMPH1 in vitro.	p. 4
Appendix Figure S2 - Replicate comparison of MS1 peak areas.	p. 5
Appendix Figure S3 - MS1 precursor ion chromatograms.	p. 6
Appendix Figure S4 - ARHGEF2 and EB2 are efficiently phosphorylated by CDKL5 in vitro.	p. 7
Appendix Figure S5 - MAP1S LC S786/812D is not phosphomimetic.	p. 8
Appendix Figure S6 - IPSC pluripotency markers.	p. 9

Appendix Figure S1 - Analog-sensitive CDKL5 phosphorylates AMPH1 *in vitro*.

A CDKL5 gatekeeper residue identified by sequence alignment with kinase domains of NDR1 and MST3. Gatekeeper mutations are highlighted in bold.

B CDKL5 C152A rescue mutation was determined by sequence alignment with NDR1 S229A rescue mutation. Both are at -1 of conserved DFG motif of the kinase domain. Rescue mutations are highlighted in bold.

C *In vitro* kinase assay showing that 50 ng (40 nM) AS-CDKL5 directly phosphorylates 300 ng (130 nM) AMPH1 in 60 minutes incubation. Thio-P = anti-thiophosphate ester antibody.

Appendix Figure S2 – Replicate comparison of MS1 peak areas.

A - C Peak areas of precursor ion chromatograms are extracted after MS1 filtering for peaks picked based on MS2 peptide identification in three independent experiments. Peaks for all three putative CDKL5 substrate peptides were found in experiment 1 (A), but only ARHGEF2 and EB2 peaks were present in experiment 2 (B) and 3 (C). Peak areas are averaged for multiple injections (n = 2 for experiment 1 and 2, n = 3 for experiment 3). Isotope dot product is depicted above the respective bars.

Appendix Figure S3 - MS1 precursor ion chromatograms.

A - E Precursor ion chromatograms extracted after MS1 filtering for peaks picked based on MS2 peptide identification in experiment 1. Black arrowheads point towards peaks for ARHGEF2 (A), EB2 double charged (B) and triple charged (C), and MAP1S double charged (D) and triple charged (E) precursor ions. Retention time and mass error are depicted above the respective peaks.

Appendix Figure S4 - ARHGEF2 and EB2 are efficiently phosphorylated by CDKL5 *in vitro*.

A - D *In vitro* kinase assays showing efficient ARHGEF2 phosphorylation by CDKL5. 50 ng (40 nM) AS-CDKL5 phosphorylates 150 ng (50 nM) ARHGEF2 (A, B). In 60 minutes of incubation, 150 ng ARHGEF2 is phosphorylated by small amounts of CDKL5 (C, D). Quantification of phosphorylated ARHGEF2 is normalized to maximum intensity.

E - H *In vitro* kinase assays showing efficient EB2 phosphorylation by CDKL5. 50 ng (40 nM) AS-CDKL5 phosphorylates 200 ng (170 nM) EB2 very rapidly (E, F). In 30 minutes of incubation, 200 ng

EB2 is phosphorylated by small amounts of CDKL5 (G, H). Quantification of phosphorylated ARHGEF2 is normalized to maximum intensity.

Appendix Figure S5 - MAP1S LC S786/812D is not phosphomimetic.

A, B Taxol-stabilised microtubule co-sedimentation assay with a 1:1 MT:MAP1S LC ratio shows a higher percentage of soluble MAP1S LC (48 ± 5 %). MAP1S pS786 and pS812 are not restricted to the soluble or MT-bound fraction. Quantification of MAP1S phosphorylation is normalized for total protein level. Student's t-test: n = 4 replicates.

C Taxol-stabilised microtubule co-sedimentation assay including MAP1S LC S786/812D. MAP1S LC S786/812D in the soluble supernatant (s/n) fraction is reduced compared to WT MAP1S LC and therefore does not act like a phosphomimetic variant.

D COS-7 cell expressing HA-MAP1S LC S786/812D is shown with endogenous α-tubulin staining. MAP1S LC localizes to microtubules and is not phosphomimetic. Scale bar is 20 µm.

Appendix Figure S6 - IPSC pluripotency markers.

Representative images showing the expression of pluripotency markers OCT4 and TRA-1-60 in human CDKL5-mutant and control IPSCs. Scale bar is 100 µm.

Appendix Figure S1

A

Gatekeeper mutation							
CDKL5	Y	L	V	**F89A**	E	F	M
NDR1	Y	L	I	**M166A**	E	F	L
MST3	W	I	I	**M99I**	E	Y	L

B

Rescue mutation						
CDKL5	K	L	**C152A**	D	F	G
NDR1	K	L	**S229A**	D	F	G

C

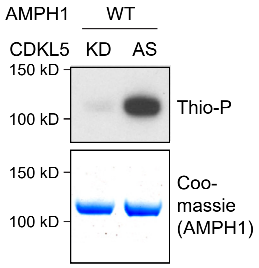

Appendix Figure S2

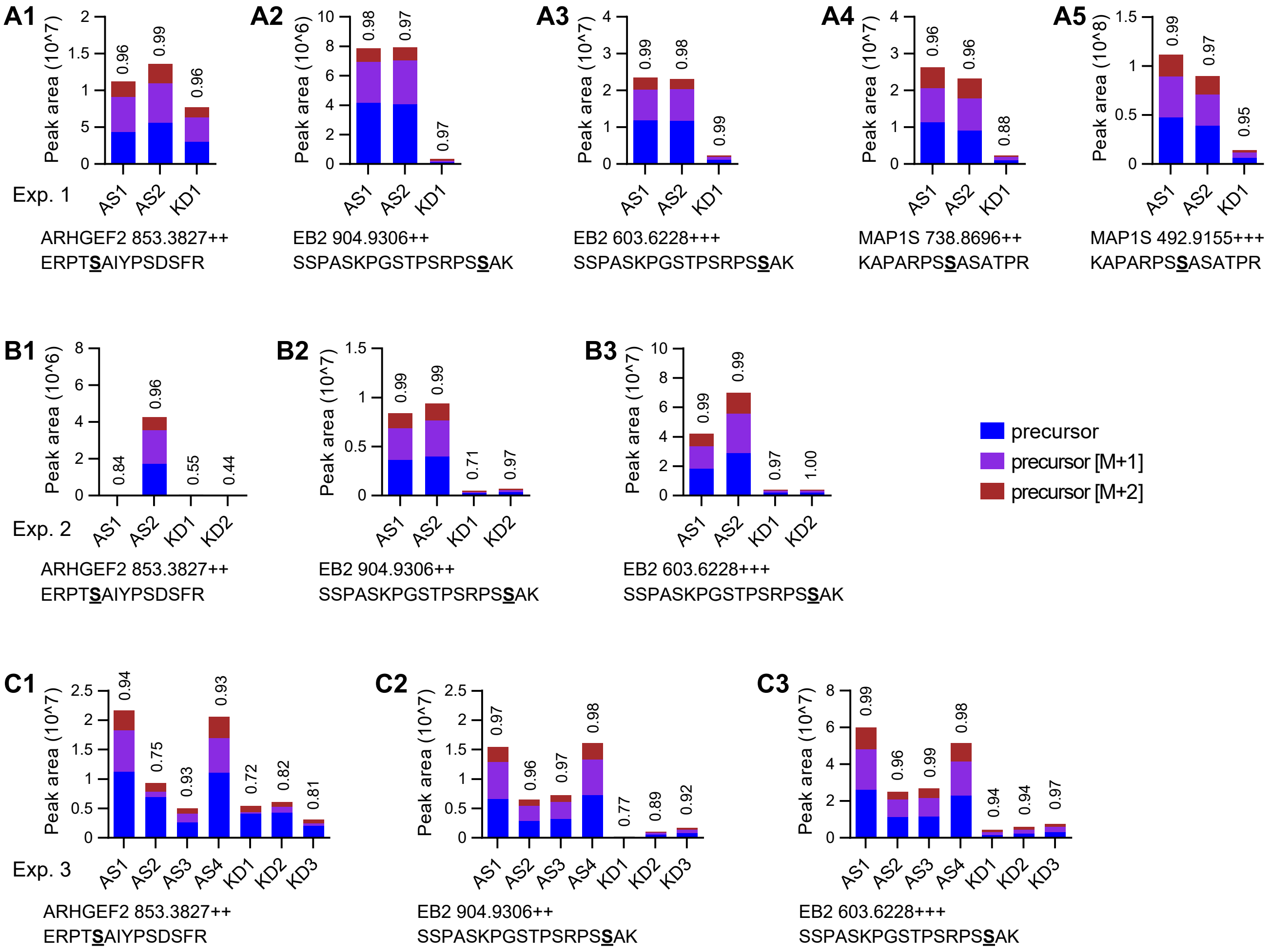

Appendix Figure S3

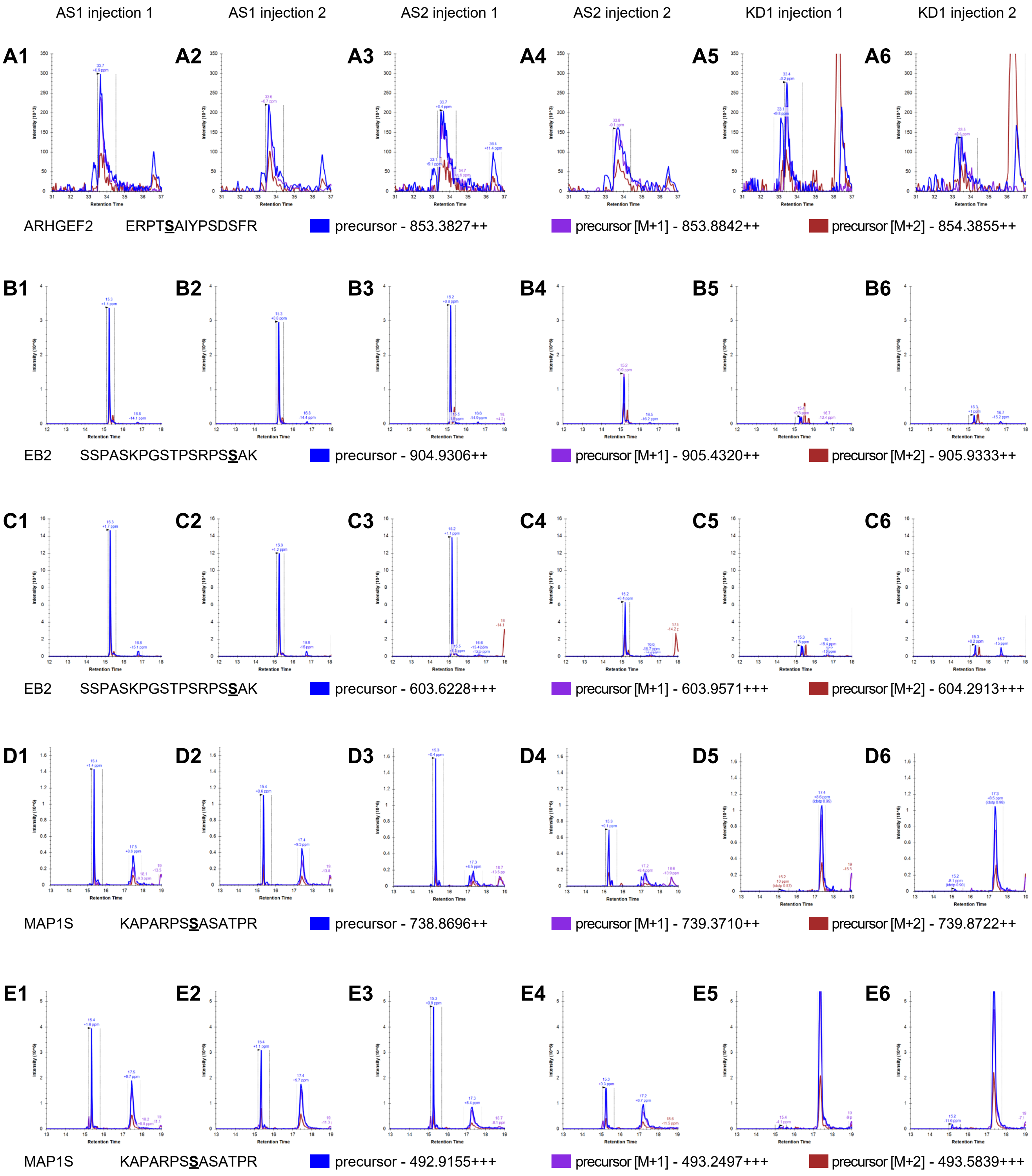

Appendix Figure S4

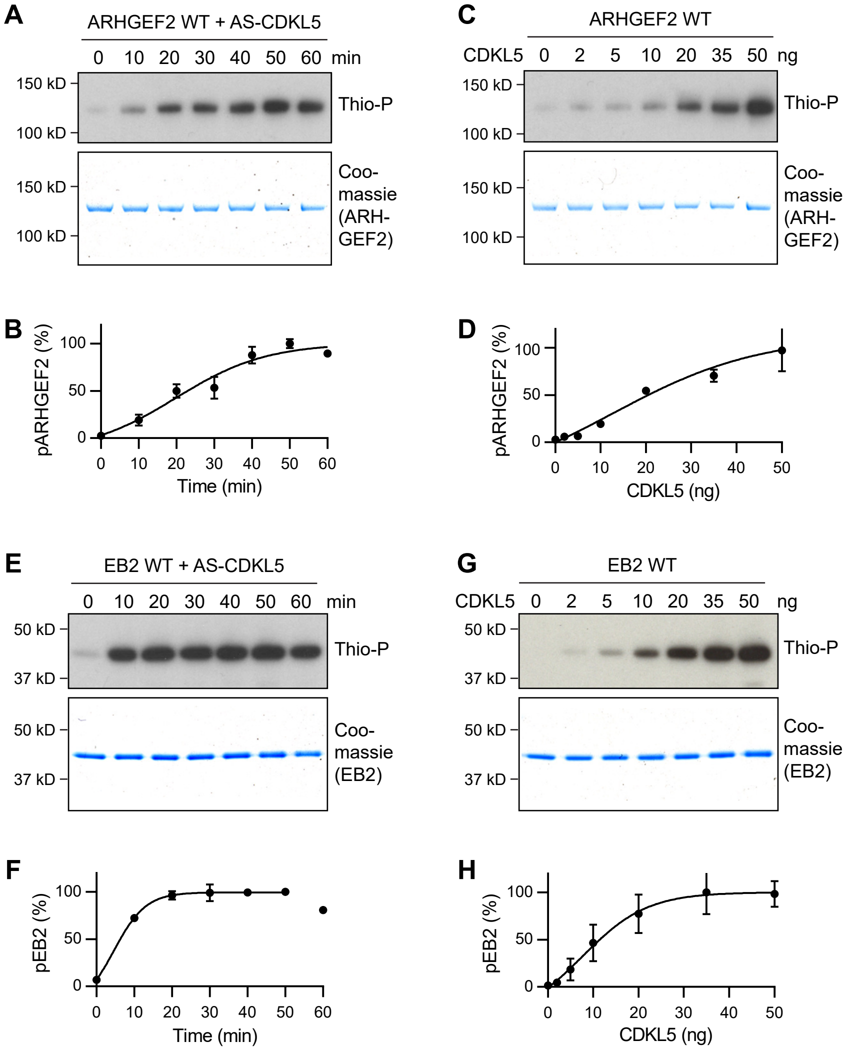

Appendix Figure S5

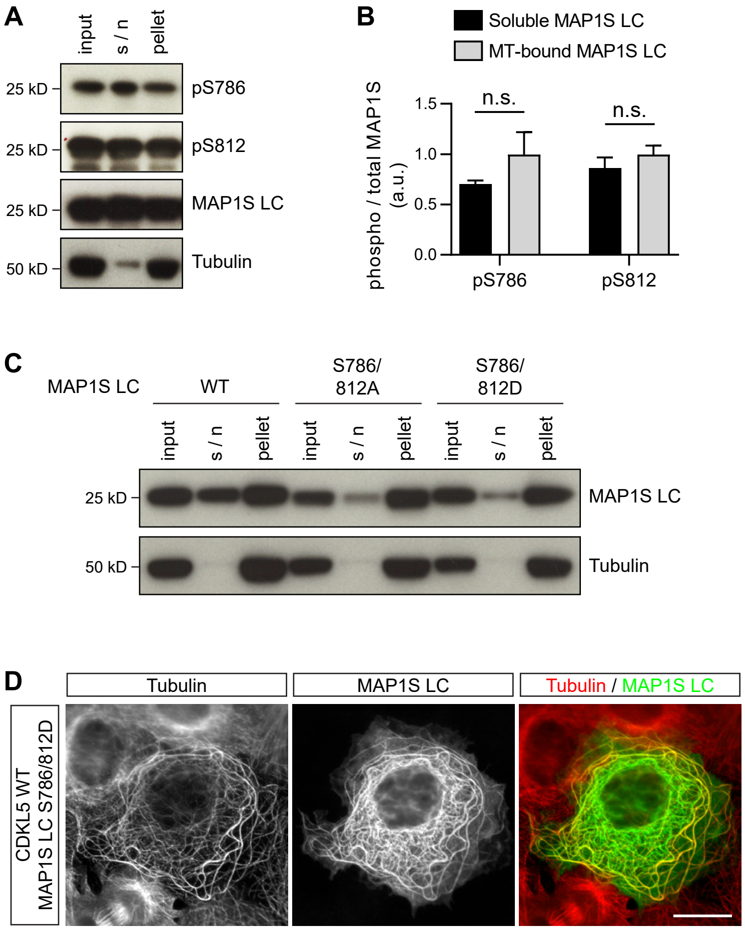

Appendix Figure S6

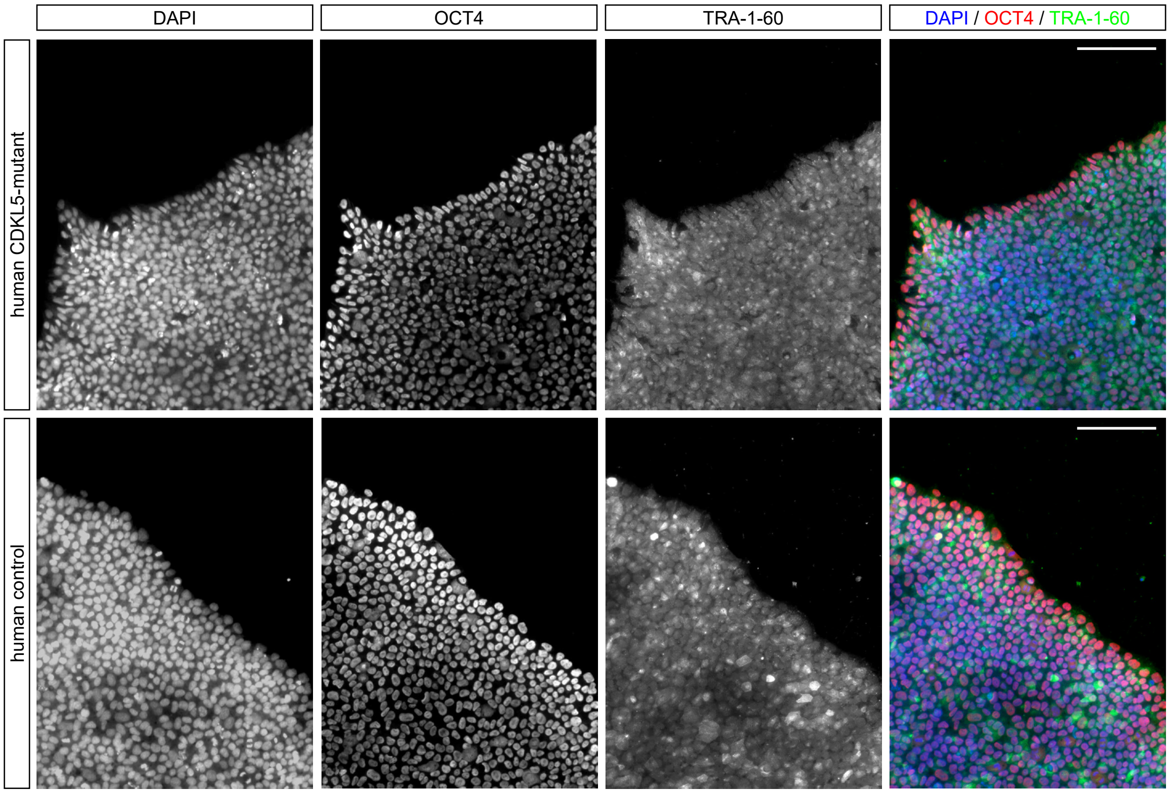

Supplement: Supplementary file 1 — Appendix [file EMBJ-37-e99763-s001.pdf]
