# Supplementary material for: Chemical genetic identification of CDKL5 substrates reveals its role in neuronal microtubule dynamics
Source: EMBO J. 2018 Sep 28;37(24):e99763. doi: 10.15252/embj.201899763 (PMC6293278; doi:10.15252/embj.201899763)
Supplement: Supplementary file 2 — Expanded View Figures PDF [file EMBJ-37-e99763-s002.pdf]

Expanded View Figures

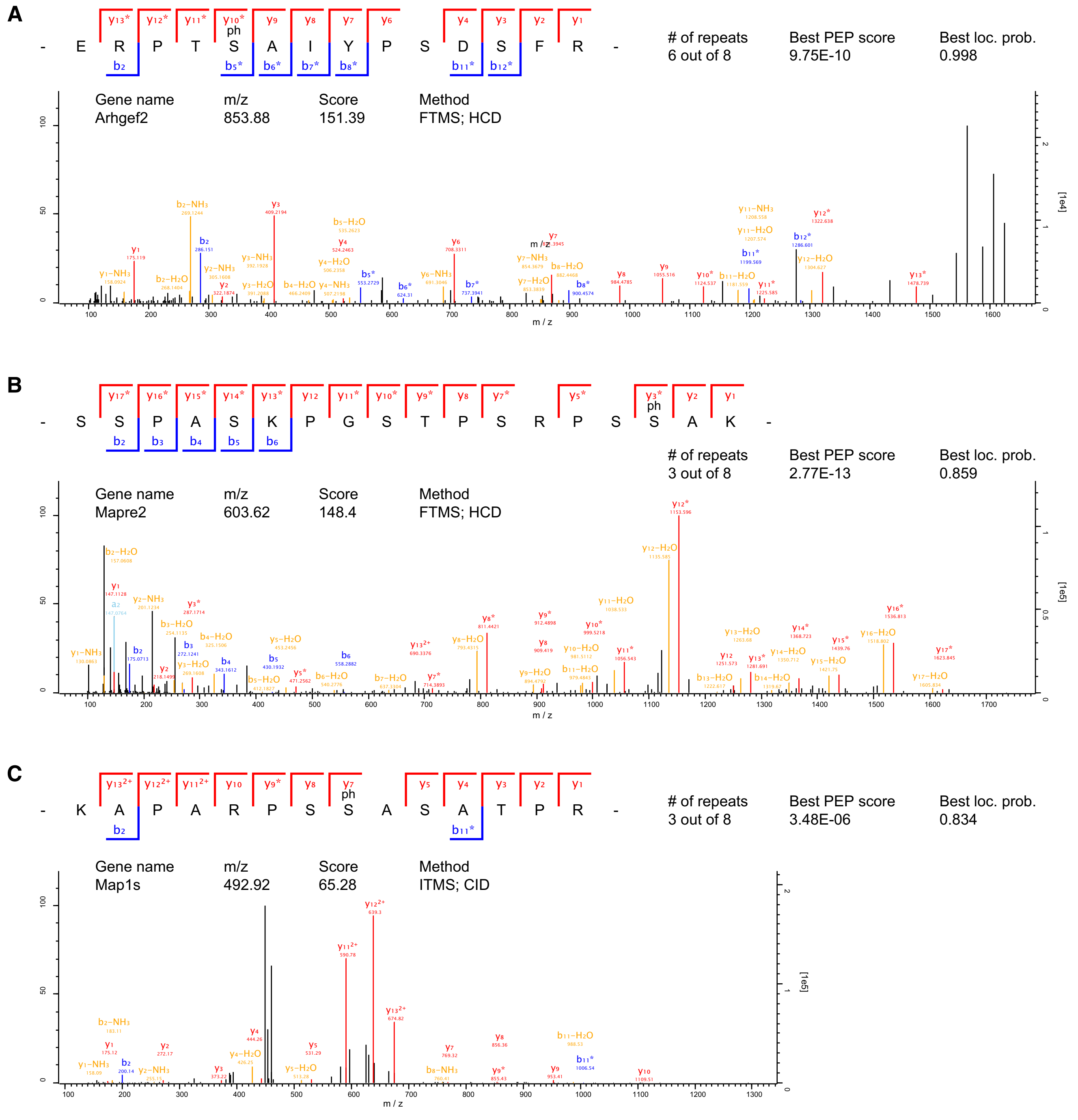

Figure EV1. MS2 spectra of CDKL5 substrates.

A–C Best identification spectra for ARHGEF2 (A), EB2 (B) and MAP1S (C) isolated by chemical genetic covalent capture for CDKL5 substrates. Sequences are determined by product ions of b- and y-series after collision-induced dissociation (CID) or higher-energy C-trap dissociation (HCD). Neutral loss of phosphoric acid generates a product ion series that corresponds only to those ions containing the phosphorylated residue (*). # of repeats represent the number of CDKL5 AS samples in which the specific phosphosite was identified. Only phosphosites never identified in KD samples were considered putative CDKL5 targets.

© 2018 The Francis Crick Institute

Figure EV2. Validation of phosphospecific antibodies.

A–C HEK293 cells co-transfected with full-length WT or KD FLAG-CDKL5 and Strep-ARHGEF2 (A), HA-EB2 (B) or HA-MAP1S (C) are probed with their respective phosphospecific antibodies. Phosphospecific antibodies do not detect phosphomutants, indicating their specificity. ARHGEF2 pS122 (A) and MAP1S pS786 (C) are increased when WT CDKL5 is expressed. High levels of endogenous EB2 pS222 (B) and MAP1S pS812 (C) are not altered with WT CDKL5. Total levels of kinase (FLAG) and substrate (Strep/HA) are detected by epitope tags.

D, E Full molecular weight range of Western blots with mouse P20 cortical lysates probed for total EB2 and EB2 pS222 (D) and MAP1S light chain and MAP1S pS812 (E).

F Efficient shRNA-mediated knockdown of EB2 in rat primary neurons is shown by the specific loss of EB2 staining in transfected cells. Scrambled shRNA was used as a control. EB2 pS222 signal is apparent in dendrites of the control (arrowheads). The remaining nuclear signal of EB2 pS222 after shRNA-mediated knockdown (*) is considered non-specific. Scale bar is 10 μm.

Source data are available online for this figure.

© 2018 The Francis Crick Institute

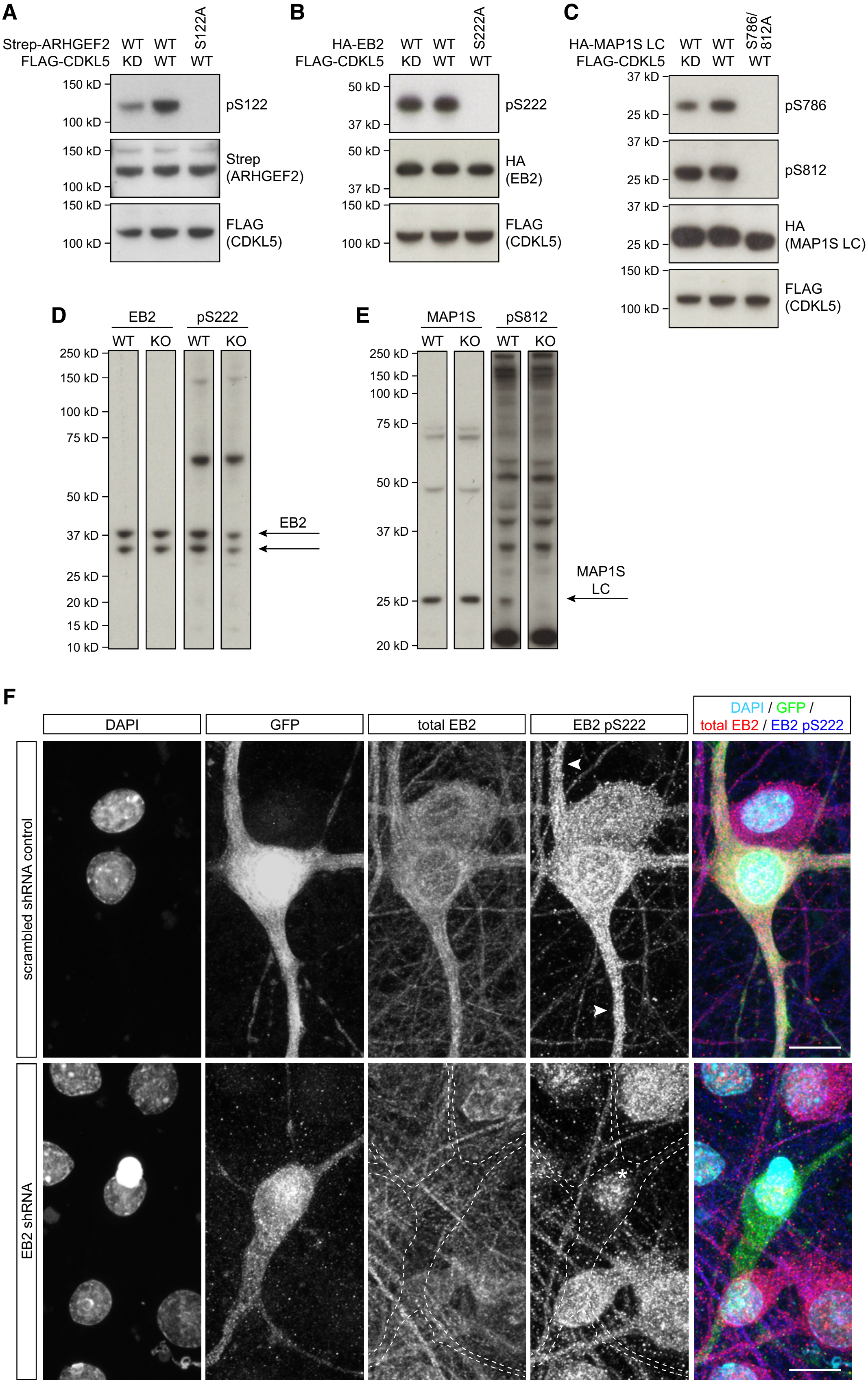
A
Strep-ARHGEF2 WT WT S122A
FLAG-CDKL5 KD WT WT
150 kD
100 kD
pS122
Strep (ARHGEF2)
FLAG (CDKL5)
B
HA-EB2 WT WT S222A
FLAG-CDKL5 KD WT WT
50 kD
37 kD
pS222
HA (EB2)
FLAG (CDKL5)
C
HA-MAP1S LC WT WT S786/812A
FLAG-CDKL5 KD WT WT
37 kD
25 kD
pS786
pS812
HA (MAP1S LC)
FLAG (CDKL5)
D
EB2 pS222
WT KO WT KO
250 kD
150 kD
100 kD
75 kD
50 kD
37 kD
25 kD
20 kD
15 kD
10 kD
EB2
E
MAP1S pS812
WT KO WT KO
MAP1S LC
F
DAPI
GFP
total EB2
EB2 pS222
DAPI / GFP / total EB2 / EB2 pS222
scrambled shRNA control
EB2 shRNA

Figure EV2.

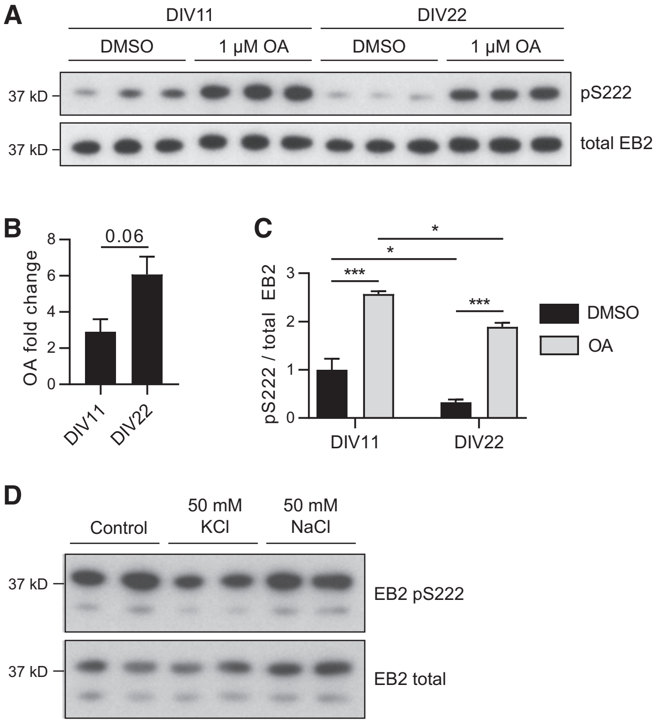

Figure EV3. Changes in EB2 phosphorylation are not due to phosphatases or osmolarity.

A–C Phosphatase activity does not attribute to the reduced EB2 phosphorylation in mature neurons. EB2 phosphorylation is reduced in mature rat primary cortical neurons at DIV22 compared to developing neurons at DIV11. Thirty-minute treatment with 1 μM okadaic acid (OA) inhibits phosphatases and increases EB2 pS222 signal. The fold change in EB2 pS222 after OA at DIV22 is trending to be larger than at DIV11 (B), but reduced EB2 phosphorylation at DIV22 is not completely rescued by phosphatase inhibition (C). Quantification of EB2 phosphorylation is normalized for total protein level. Student's *t*-test (B), Tukey's multiple comparison (C): $n = 3$ repeats, *$P < 0.05$, ***$P < 0.001$, error bars are SEM.

D NaCl treatment is a control for the changes in osmolarity induced by KCl without the depolarizing effect. Thirty-minute treatment of rat primary cortical neurons with 50 mM NaCl did not lead to the reduction in EB2 S222 phosphorylation observed with the same amount of KCl.

Source data are available online for this figure.

© 2018 The Francis Crick Institute

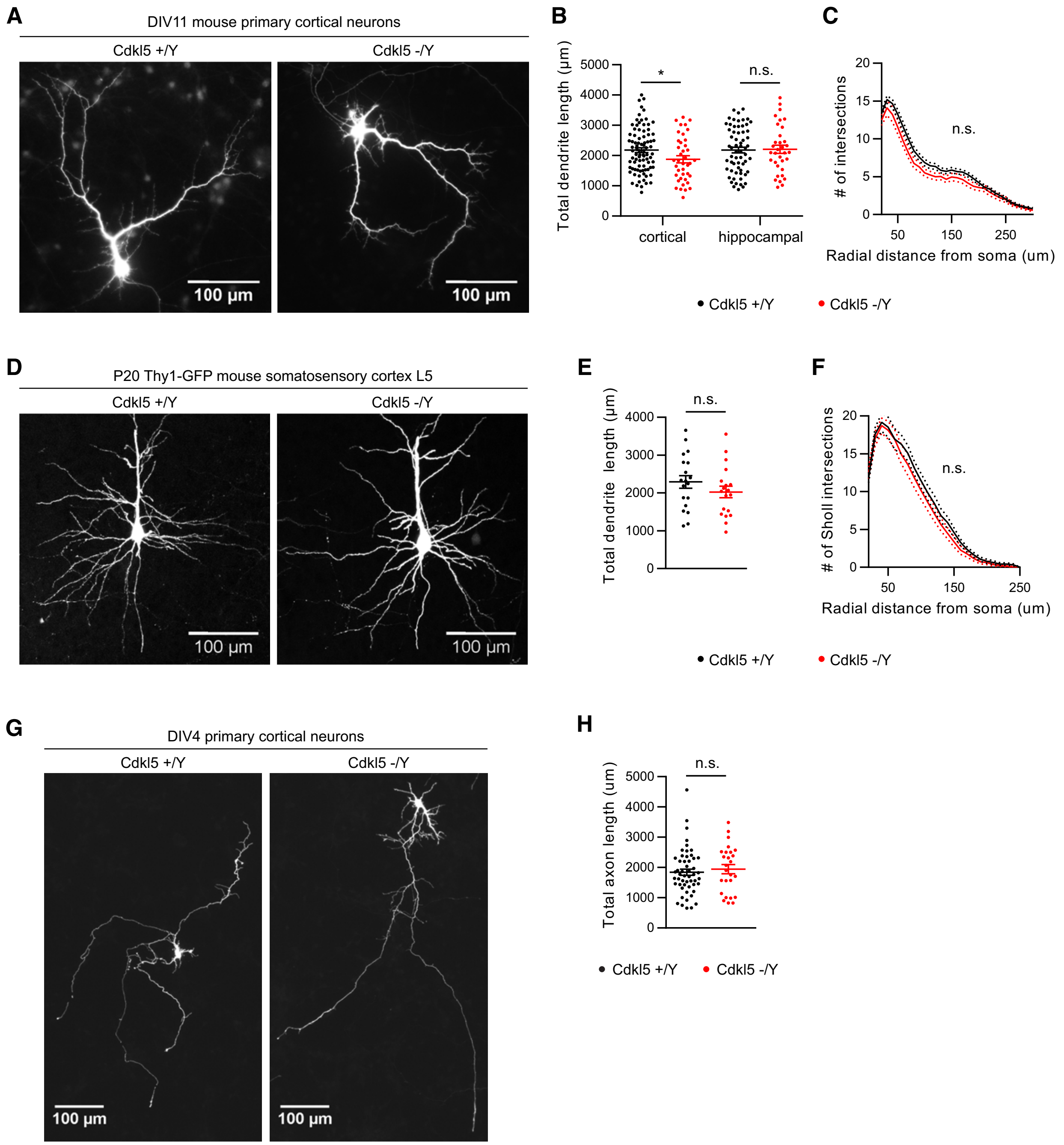

Figure EV4. Morphological characterization of dendrites and axons in CDKL5 KO neurons.

A–C Primary cortical or hippocampal neuron cultures are prepared from CDKL5^{+}/Y and CDKL5^{-}/Y littermate embryos. Neurons are transfected with GFP at DIV7 and fixed at DIV11 to analyse dendrite morphology. Representative images of cortical neurons are shown (A). Total dendrite length is slightly reduced in cortical neurons, but not in Ctip2-negative hippocampal CA3 neurons (B). Sholl analysis of cortical neurons does not reveal specific compartmentalization (C). Student's *t*-test: n = 2–4 animals, 34–63 neurons.

D–F Layer V pyramidal neuron basal dendrites are examined using thy1-YFP CDKL5^{+}/Y and CDKL5^{-}/Y mice. Representative images for *z*-projected dendrites are shown (D). Dendrite length (E) or Sholl analysis (F) is not different between WT and KO mice. Student's *t*-test: n = 4 animals, 19 neurons per genotype.

G, H Primary cortical neuron cultures are prepared from CDKL5^{+}/Y and CDKL5^{-}/Y littermate embryos. Neurons are transfected with GFP at DIV2 and fixed at DIV4 to analyse axon morphology. Representative images of cortical neurons are shown (G). Total axon length is not significantly different between CDKL5 WT and KO neurons (H). Student's *t*-test: n = 2–4 animals, 26–52 neurons.

Data information: Dotted lines in (C, F) represent error bars. n.s., not significant, *P < 0.05, error bars are SEM.

© 2018 The Francis Crick Institute

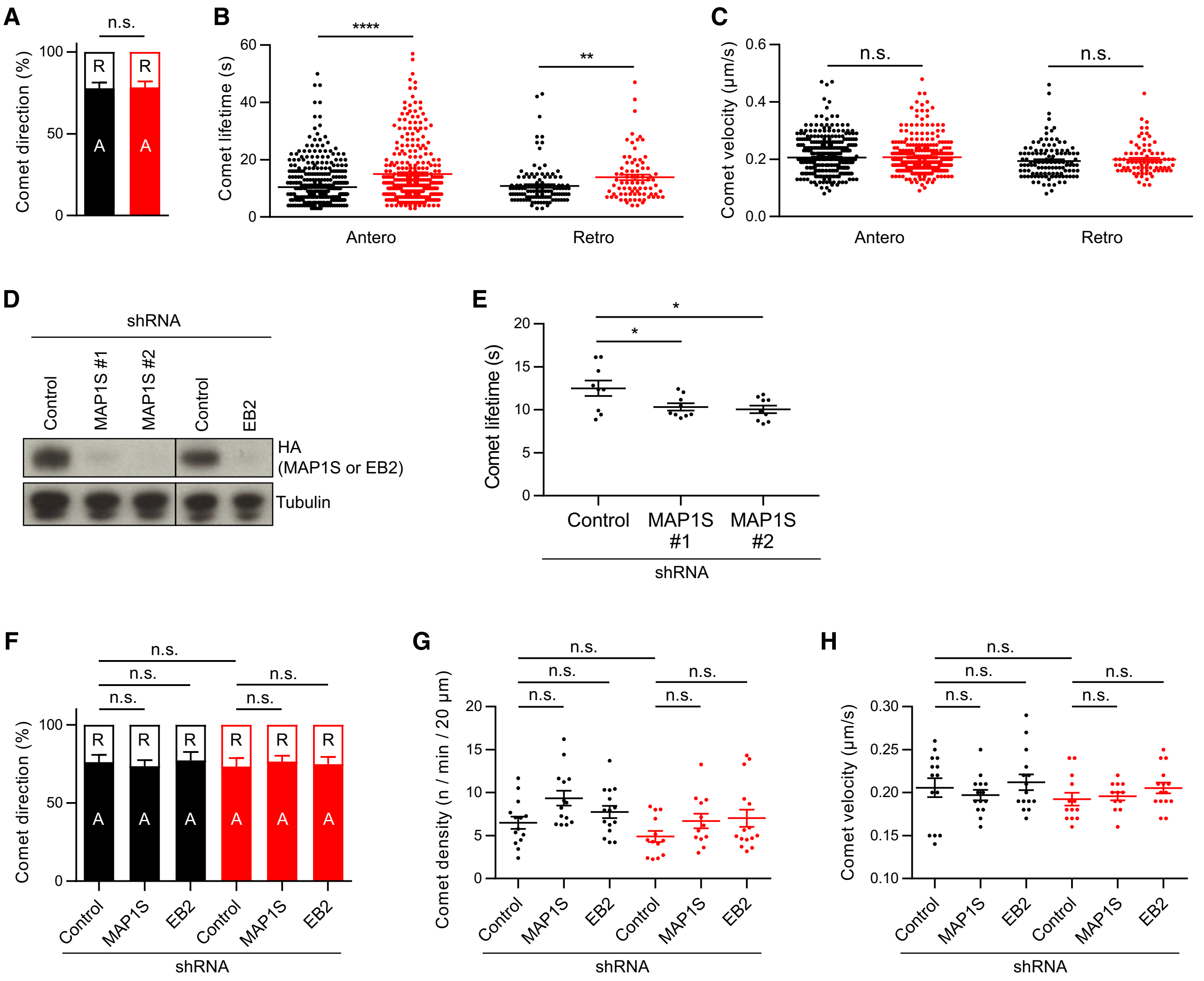

Figure EV5. CDKL5 alters microtubule dynamics via MAP1S.

A–C CDKL5-dependent microtubule dynamics are not dependent on direction. EB3-tdTomato comet direction is not altered in CDKL5 KOs (A). Comet lifetime is increased in both anterograde and retrograde comets of KO neurons (B). Comet velocity is not changed in CDKL5 KOs when anterograde and retrograde comets are analysed separately (C). Student's *t*-test: n = 15–16 neurons, 302–496 anterograde comets, 80–120 retrograde comets.

D shRNA-mediated knockdown showing reduction in rat HA-MAP1S and mouse HA-EB2 overexpressed in HEK293 cells.

E Two independent shRNAs targeting MAP1S cause reduction in comet lifetime. Dunnett's multiple comparison: n = 7–8 neurons, 284–402 comets.

F–H EB3-tdTomato comet direction (F), density (G) and velocity (H) are unchanged in MAP1S or EB2 shRNA-expressing mouse cortical cultures. Tukey's multiple comparison: n = 12–15 neurons, 388–741 comets per condition. Black indicates CDKL5 WT and red indicates CDKL5 KO.

Data information: A, anterograde; R, retrograde; n.s., not significant, $^*P < 0.05$, $^{**}P < 0.01$, $^{****}P < 0.0001$, error bars are SEM.
Source data are available online for this figure.

© 2018 The Francis Crick Institute
