# Supplementary material for: Chemical genetic identification of CDKL5 substrates reveals its role in neuronal microtubule dynamics
Source: EMBO J. 2018 Sep 28;37(24):e99763. doi: 10.15252/embj.201899763 (PMC6293278; doi:10.15252/embj.201899763)
Supplement: Supplementary file 3 — Movie EV1 [file EMBJ-37-e99763-s003.zip › Movie_EV1.docx]

**Movie EV1 - EB3-tdTomato comets in WT dendrite.**

Representative video of EB3-tdTomato overexpressed in a WT mouse primary cortical neuron dendrite. Anterograde is to the right. 10 frames/s.
