# Supplementary material for: Chemical genetic identification of CDKL5 substrates reveals its role in neuronal microtubule dynamics
Source: EMBO J. 2018 Sep 28;37(24):e99763. doi: 10.15252/embj.201899763 (PMC6293278; doi:10.15252/embj.201899763)
Supplement: Supplementary file 5 — Movie EV3 [file EMBJ-37-e99763-s005.zip › Movie_EV3.docx]

**Movie EV3 - TrkB-RFP trafficking in WT dendrite.**

Representative video of TrkB-RFP overexpressed in a WT mouse primary cortical neuron dendrite. Anterograde is to the right. 6 frames/s.
