# Supplementary material for: Chemical genetic identification of CDKL5 substrates reveals its role in neuronal microtubule dynamics
Source: EMBO J. 2018 Sep 28;37(24):e99763. doi: 10.15252/embj.201899763 (PMC6293278; doi:10.15252/embj.201899763)
Supplement: Supplementary file 6 — Movie EV4 [file EMBJ-37-e99763-s006.zip › Movie_EV4.docx]

**Movie EV4 - TrkB-RFP trafficking in CDKL5 KO dendrite.**

Representative video of TrkB-RFP overexpressed in a CDKL5 KO mouse primary cortical neuron dendrite. Anterograde is to the right. 6 frames/s.
