# Supplementary material for: Chemical genetic identification of CDKL5 substrates reveals its role in neuronal microtubule dynamics
Source: EMBO J. 2018 Sep 28;37(24):e99763. doi: 10.15252/embj.201899763 (PMC6293278; doi:10.15252/embj.201899763)
Supplement: Supplementary file 7 — Movie EV5 [file EMBJ-37-e99763-s007.zip › Movie_EV5.docx]

**Movie EV5 - TrkB-RFP tracks in WT dendrite.**

Manually tracked TrkB-RFP vesicles of Supplementary video 3 using Fiji TrackMate. Color-coded backward tracks of 10 frames are displayed.
