# Supplementary material for: Chemical genetic identification of CDKL5 substrates reveals its role in neuronal microtubule dynamics
Source: EMBO J. 2018 Sep 28;37(24):e99763. doi: 10.15252/embj.201899763 (PMC6293278; doi:10.15252/embj.201899763)
Supplement: Supplementary file 8 — Movie EV6 [file EMBJ-37-e99763-s008.zip › Movie_EV6.docx]

**Movie EV6 - TrkB-RFP tracks in CDKL5 KO dendrite.**

Manually tracked TrkB-RFP vesicles of Supplementary video 3 using Fiji TrackMate. Color-coded backward tracks of 10 frames are displayed.
