# Supplementary figures and images for: Chemical genetic identification of CDKL5 substrates reveals its role in neuronal microtubule dynamics
Source: EMBO J. 2018 Sep 28;37(24):e99763. doi: 10.15252/embj.201899763 (PMC6293278; doi:10.15252/embj.201899763)

**EV2A**ARHGEF2  
pS122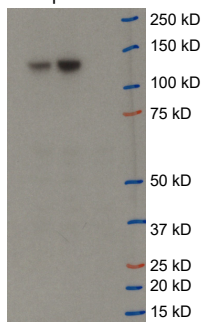

Strep

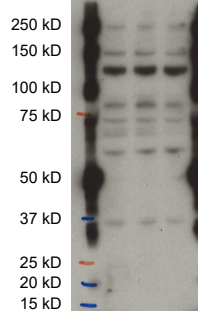**EV2B**

HA

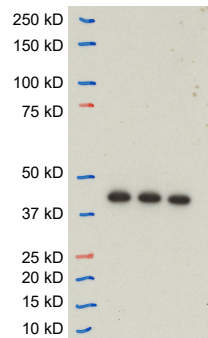**EV2C**MAP1S  
pS786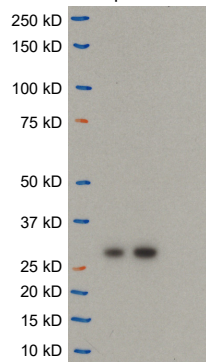MAP1S  
pS812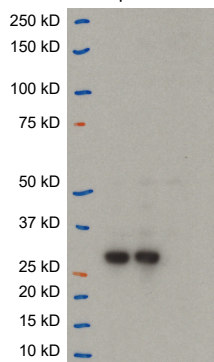

HA

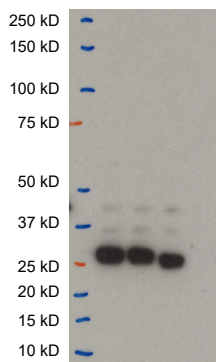**EV2A-C**

FLAG

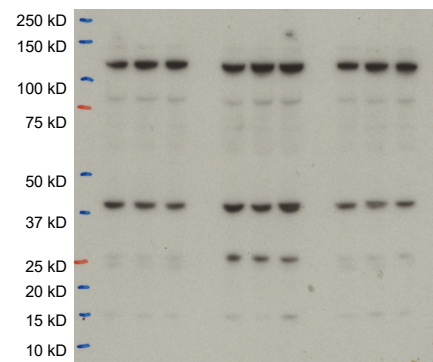

Supplement: Supplementary file 9 — Source Data for Expanded View [file EMBJ-37-e99763-s014.zip › Figure_EV2_Source_Data.pdf]

**EV3A**

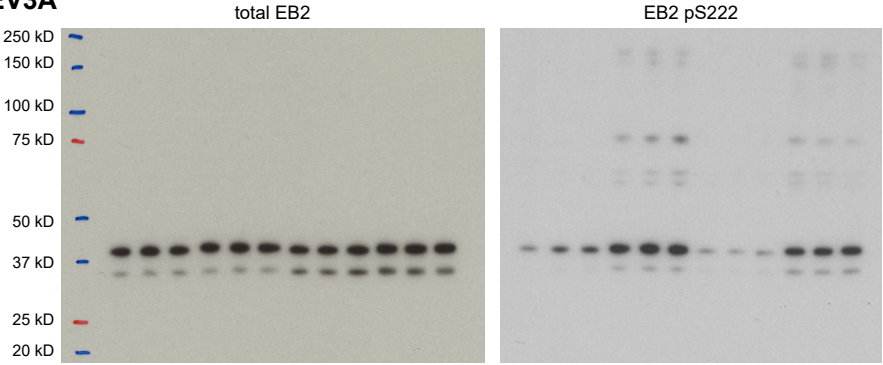

**EV3D**

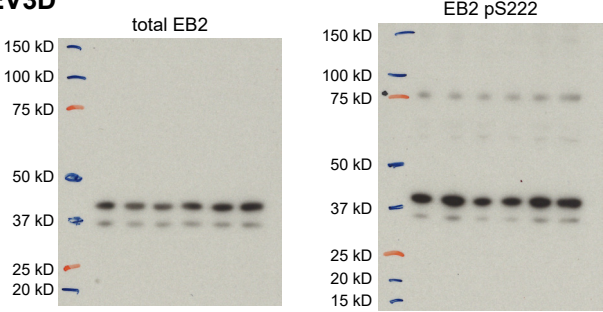

Supplement: Supplementary file 9 — Source Data for Expanded View [file EMBJ-37-e99763-s014.zip › Figure_EV3_Source_Data.pdf]

**EV5D**

HA and Tubulin

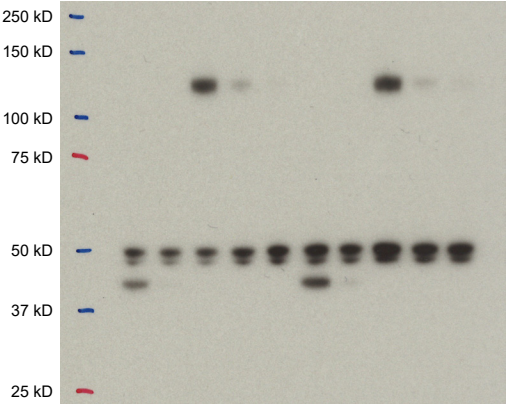

Supplement: Supplementary file 9 — Source Data for Expanded View [file EMBJ-37-e99763-s014.zip › Figure_EV5_Source_Data.pdf]

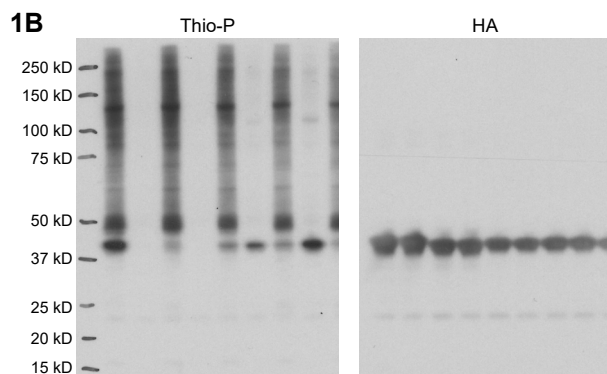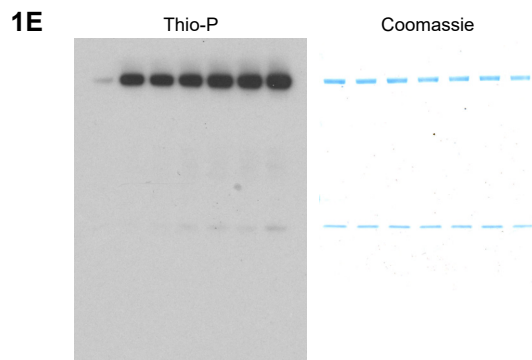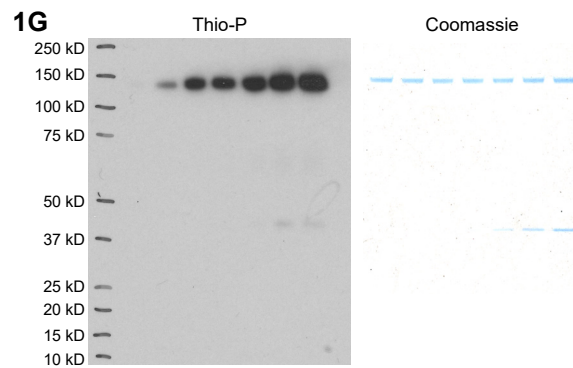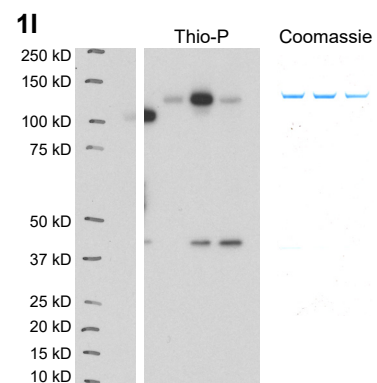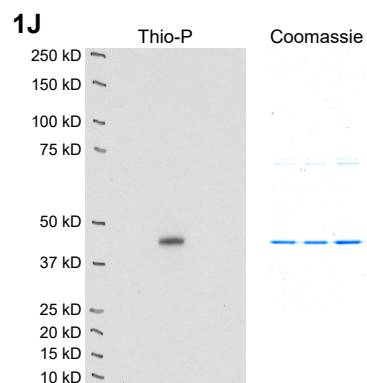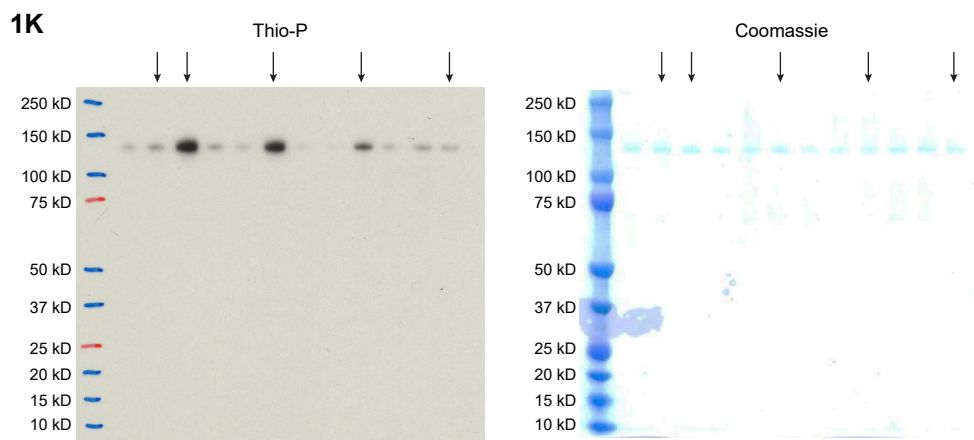

Arrows indicate lanes used for figure

Supplement: Supplementary file 11 — Source Data for Figure 1 [file EMBJ-37-e99763-s009.pdf]

**2A**

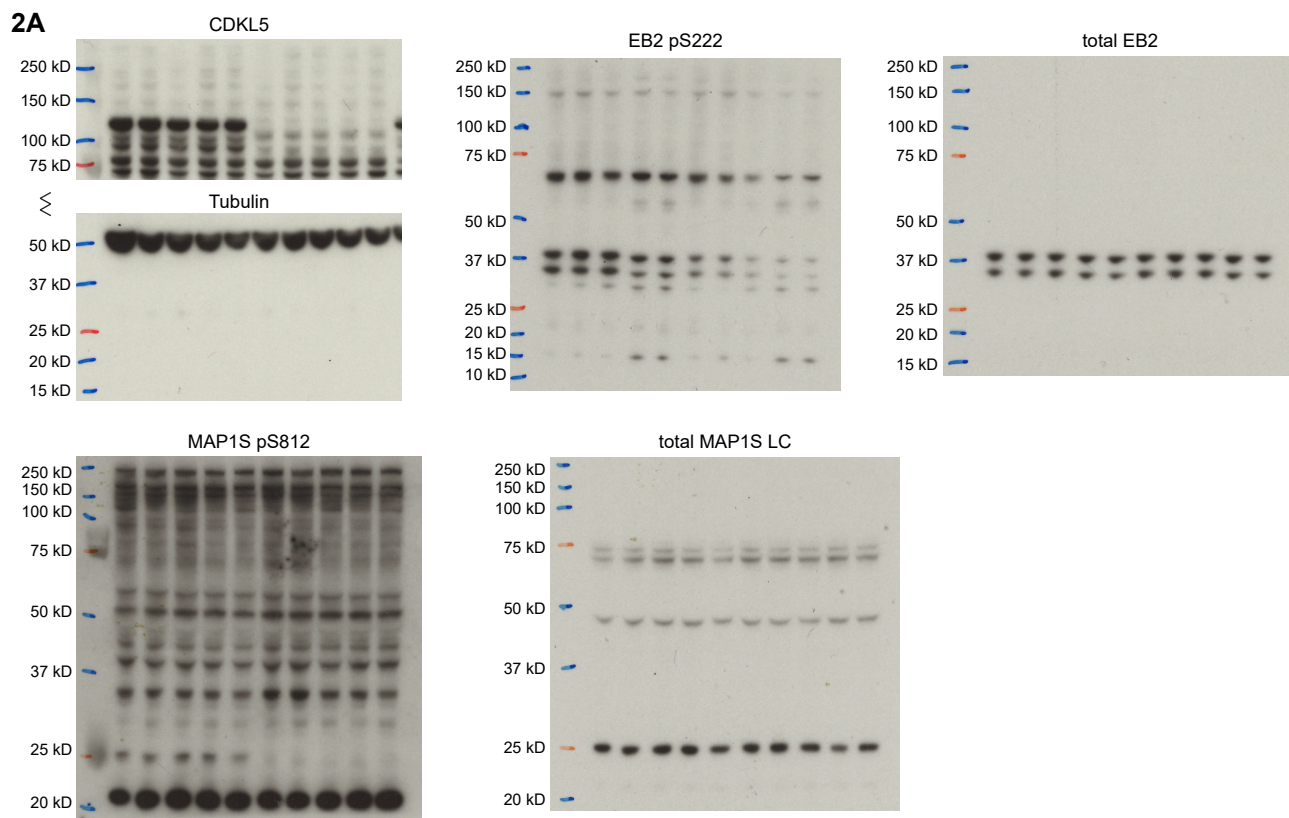

**2C**

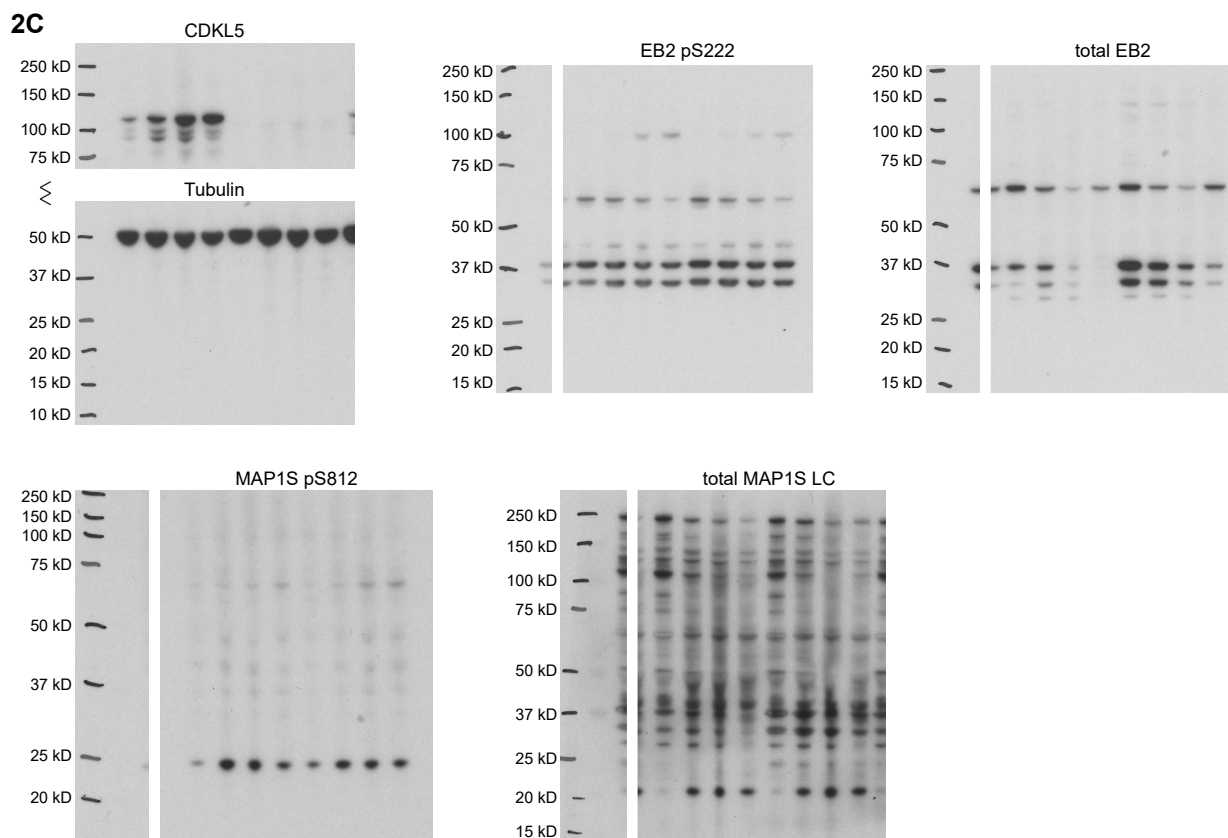

2E

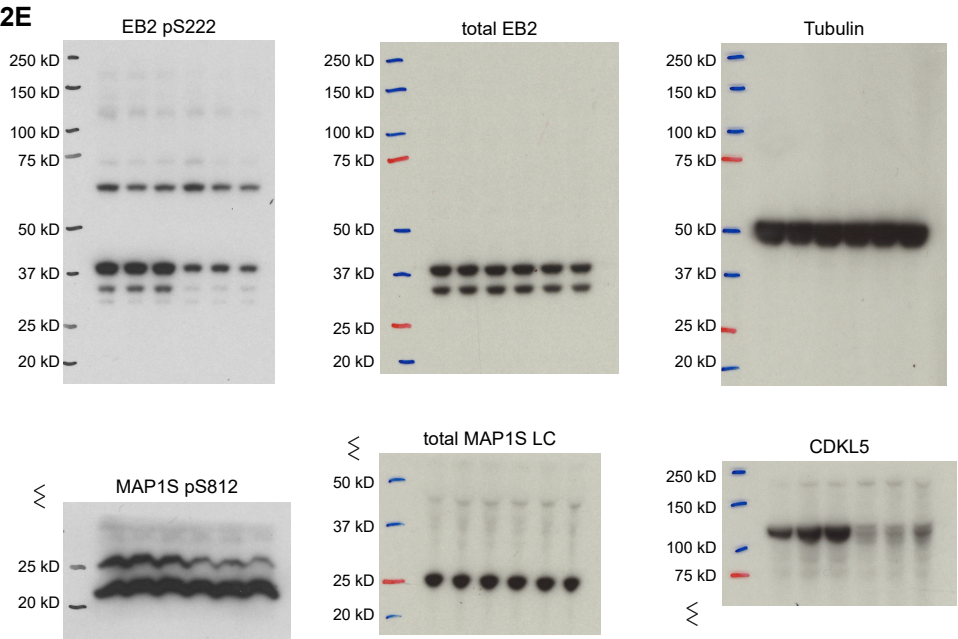

Supplement: Supplementary file 12 — Source Data for Figure 2 [file EMBJ-37-e99763-s010.pdf]

**3A**

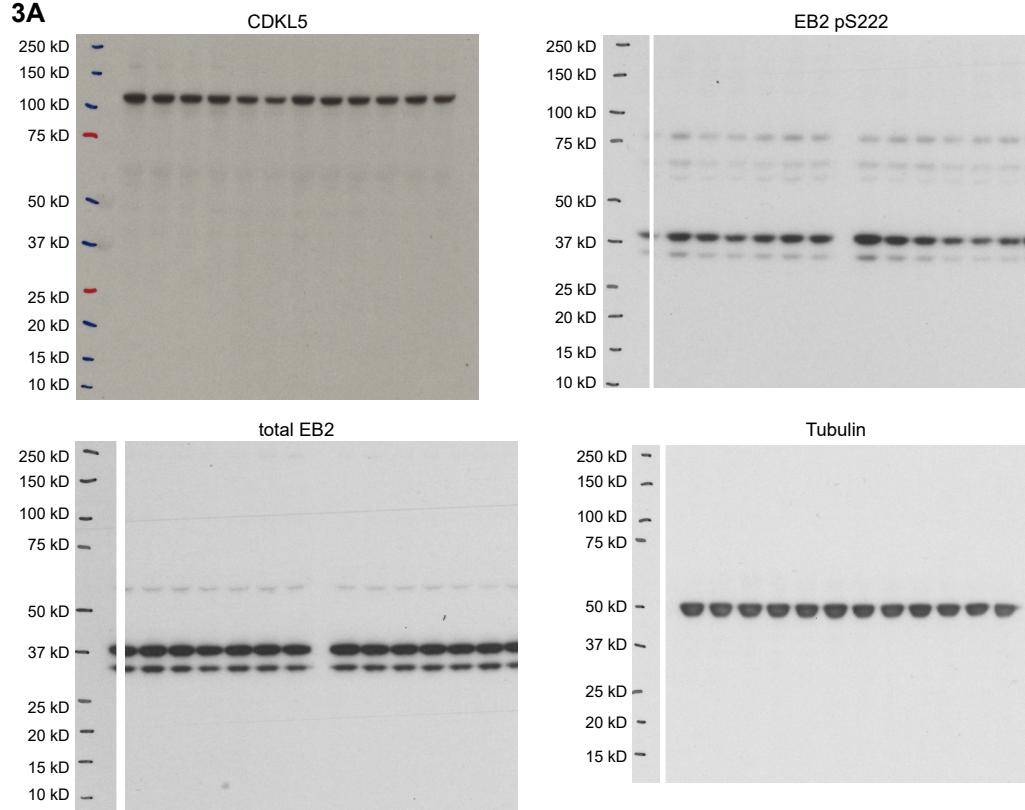

**3D**

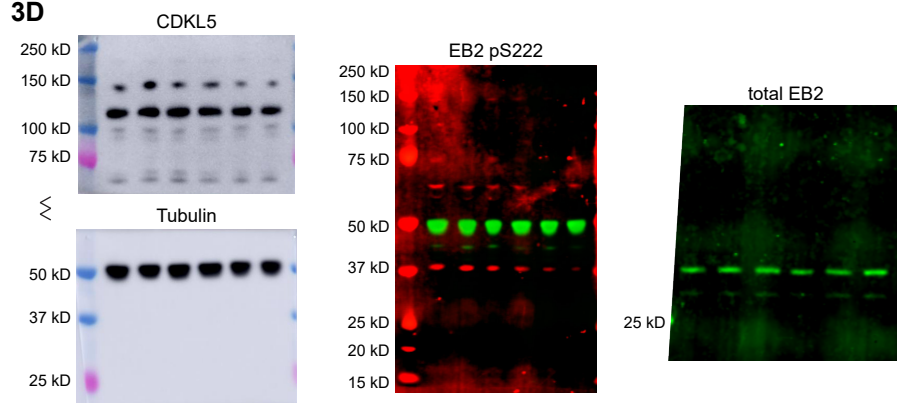

Supplement: Supplementary file 13 — Source Data for Figure 3 [file EMBJ-37-e99763-s011.pdf]

**6E**

MAP1S LC

Tubulin

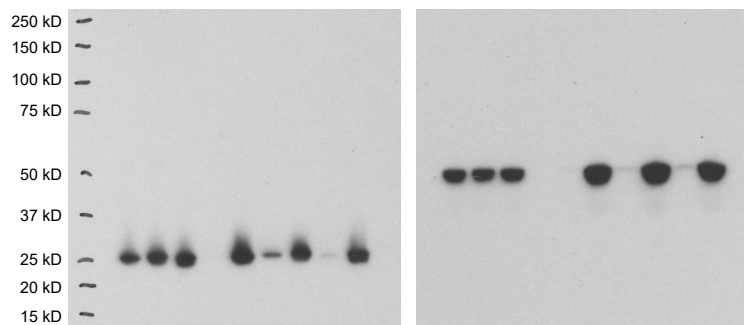

Supplement: Supplementary file 14 — Source Data for Figure 6 [file EMBJ-37-e99763-s012.pdf]

**7A**

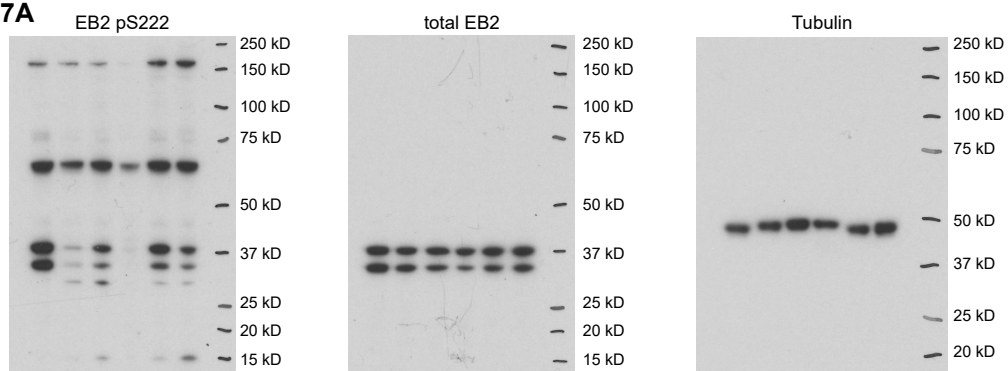

Supplement: Supplementary file 15 — Source Data for Figure 7 [file EMBJ-37-e99763-s013.pdf]
